# Acquired portosystemic collaterals: anatomy and imaging*

**DOI:** 10.1590/0100-3984.2015.0026

**Published:** 2016

**Authors:** Andréa Farias de Melo Leite, Américo Mota Jr., Francisco Abaeté Chagas-Neto, Sara Reis Teixeira, Jorge Elias Junior, Valdair Francisco Muglia

**Affiliations:** 1 PhD, Radiologist and Physician Assistant at the Instituto de Medicina Integral Professor Fernando Figueira de Pernambuco (IMIP), Maximagem, Centro Diagnóstico Lucilo Ávila Júnior, and Safelaudos, Recife, PE, Brazil.; 2 MD, Radiology Resident at the Instituto de Medicina Integral Professor Fernando Figueira de Pernambuco (IMIP), Recife, PE, Brazil.; 3 PhD, Professor in the Department of Radiology of the Universidade de Fortaleza (Unifor), Fortaleza, CE, Brazil.; 4 PhD, Pediatric Radiologist and Attending Physician at the Faculdade de Medicina de Ribeirão Preto da Universidade de São Paulo (FMRP-USP), Ribeirão Preto, SP, Brazil.; 5 PhD, Professor in the Division of Radiology of the Department of Clinical Medicine at the Faculdade de Medicina de Ribeirão Preto da Universidade de São Paulo (FMRP-USP), Ribeirão Preto, SP, Brazil.

**Keywords:** Collateral circulation, Splanchnic circulation, Hypertension, portal/complications

## Abstract

Portosystemic shunts are enlarged vessels that form collateral pathological pathways between the splanchnic circulation and the systemic circulation. Although their causes are multifactorial, portosystemic shunts all have one mechanism in common-increased portal venous pressure, which diverts the blood flow from the gastrointestinal tract to the systemic circulation. Congenital and acquired collateral pathways have both been described in the literature. The aim of this pictorial essay was to discuss the distinct anatomic and imaging features of portosystemic shunts, as well as to provide a robust method of differentiating between acquired portosystemic shunts and similar pathologies, through the use of illustrations and schematic drawings. Imaging of portosystemic shunts provides subclinical markers of increased portal venous pressure. Therefore, radiologists play a crucial role in the identification of portosystemic shunts. Early detection of portosystemic shunts can allow ample time to perform endovascular shunt operations, which can relieve portal hypertension and prevent acute or chronic complications in at-risk patient populations.

## Abstract (pt)

As vias colaterais ou *shunts* portossistêmicos são trajetos vasculares calibrosos de comunicação patológica entre a circulação esplâncnica e a sistêmica. Suas causas são multifatoriais, compartilhando um mecanismo de elevação da pressão venosa portal, a qual promove o desvio do fluxo sanguíneo do trato gastrintestinal para a circulação sistêmica. Múltiplas vias de colaterais estão descritas na literatura, sendo congênitas ou adquiridas. Ambas as causas, congênitas e adquiridas, resultam na redistribuição de volume vascular do trato gastrintestinal de veias sistêmicas e um aumento concomitante na pressão venosa portal. Os objetivos deste ensaio são: 1) discutir as características anatômicas e de imagem dos *shunts* portossistêmicos; 2) fornecer uma revisão robusta (com ilustrações e desenhos esquemáticos) para detectar e reconhecer os *shunts* portossistêmicos adquiridos. A importância do seu reconhecimento recai sobre o fato que, em alguns casos, eles são os únicos sinais que predizem a presença de hipertensão portal, sendo a avaliação do radiologista de grande valia na escolha de tratamentos endovasculares e na detecção de suas complicações.

## INTRODUCTION

Recent studies in the radiology literature of Brazil have stressed the importance of imaging in facilitating the diagnosis of hepatobiliary disorders^(1-6)^. Improvements in imaging methods have increased the importance of recognizing anomalous portosystemic shunts, especially in situations in which the signs of portal hypertension and its complications are detected by the radiologist first, even before the manifestation of clinical signs.

Portosystemic shunts, also known as portosystemic collaterals, are abnormal communications between the portal system and the systemic circulation, and such shunts can be congenital or acquired^(7,8)^. Congenital shunts can be intrahepatic or extrahepatic, and their classification is complex. The study of such shunts is beyond the scope of this essay. Rather, we have concentrated on describing and illustrating the anatomical and imaging aspects of the main drainage pathways of acquired portosystemic shunts^(9)^.

Portal hypertension secondary to chronic liver disease (cirrhosis) is the factor most often associated with portosystemic shunt. Portal obstruction can occur at postsinusoidal, sinusoidal, or presinusoidal sites, portal vein thrombosis being the major cause of prehepatic portal hypertension, as well as potentially being related to inflammation, bleeding disorders, trauma, and neoplasia^(9)^.

For a complete understanding of the pathophysiology of hepatofugal redistribution of portal blood flow, it is necessary to examine clinical and chronological data, as well as to recognize its aspects on the various imaging modalities.

## ANATOMY OF THE PORTAL VENOUS SYSTEM

**Figure 1:**
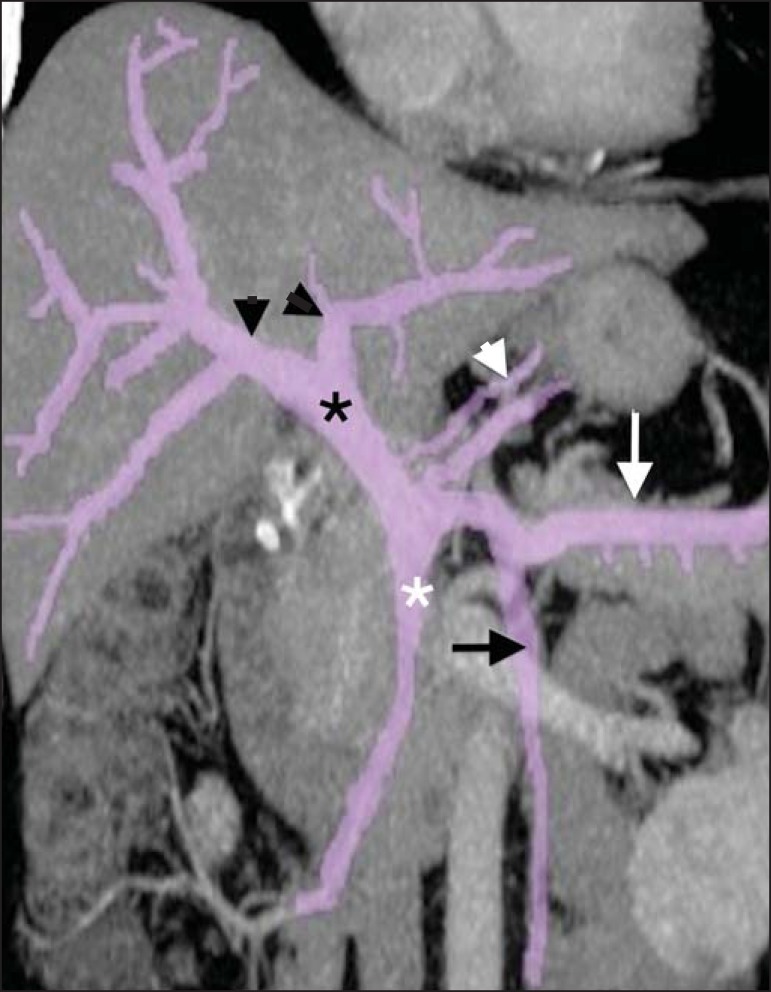
Reformatted coronal computed tomography scan showing the anatomy of the portal venous system. The main portal vein (black asterisk) is formed by the junction of the superior mesenteric vein (white asterisk) and the splenic vein (white arrow). The short gastric veins, pancreatic branches, and the gastroepiploic vein (white arrowhead) drain into the splenic vein. The right and left branches of the portal vein are indicated by black arrowheads, and the inferior mesenteric vein is indicated by a black arrow.

The portal venous system consists of vasculature that drains the digestive tract, spleen, pancreas, and biliary system. The portal vein lies in a transverse fissure, between the quadrate and caudate lobes, on the visceral surface of the liver. Typically, the portal vein arises from the junction of the superior mesenteric vein and the splenic vein, at the level of the neck of the pancreas^(10)^, as depicted in Figure 1.

The falciform ligament on the diaphragmatic surface of the liver and the round ligament on its visceral surface are the fibrous remnants of the umbilical vein, which carried blood from the placenta to the porta hepatis during the fetal period. The venous ligament corresponds to the drainage pathway for flow diverted from the umbilical vein to the inferior vena cava via the ductus venosus. In adults, these ligaments are major anatomical landmarks. The falciform ligament separates the medial segment of the left hepatic lobe (segment IV) from its lateral segments (segments II and III) on its superior surface. On the visceral surface of the liver, the round ligament marks the boundary between the quadrate lobe and the left hepatic lobe, the venous ligament, because of its posterior location, demarcating the division between the caudate lobe and the left hepatic lobe^(11)^.

**Figure 2:**
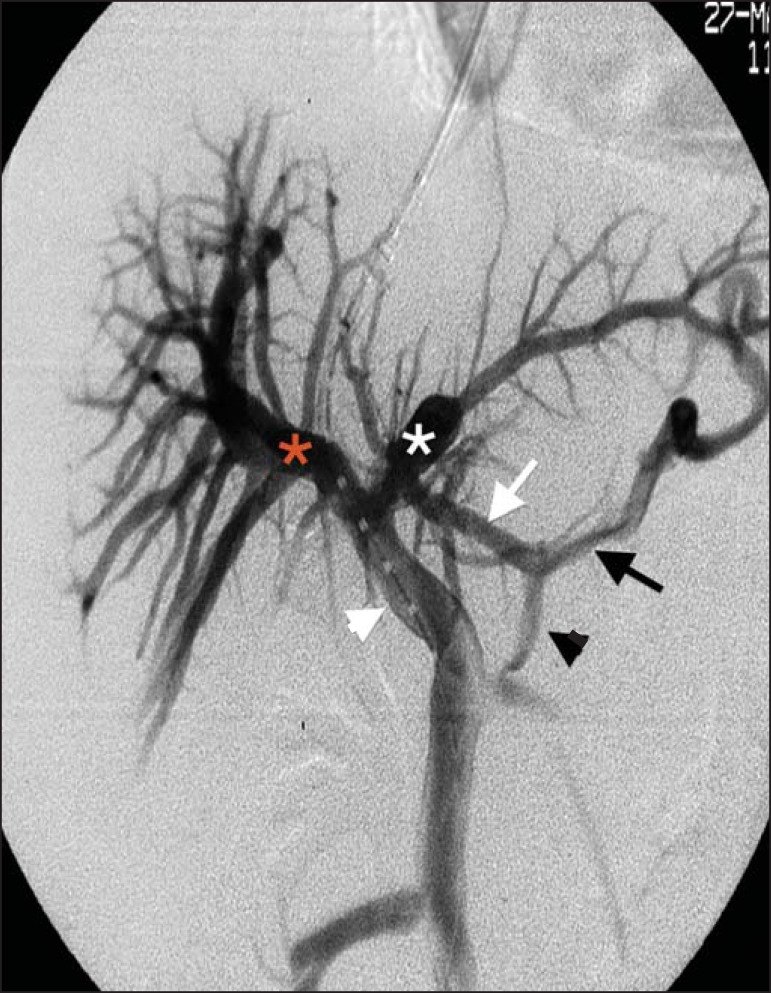
Venography showing the left gastric vein (black arrowhead), its anterior branches (white arrow), and its posterior branches (black arrow); increases in the caliber of the anterior and posterior branches lead to the formation of esophageal and paraesophageal varices, respectively. Note the main portal vein (white arrowhead), with its right and left branches (red and white asterisks, respectively).

The splenic vein has several tributaries, including vascular branches that drain the pancreas, the short gastric veins, and the left gastroepiploic vein. The superior mesenteric vein drains the right colon, the small bowel, and the pancreas, whereas the inferior mesenteric vein drains the left colon and the rectum^(10)^, as shown in Figure 2. Physiologically, the pressure in the portal system is the product of blood flow multiplied by vascular resistance (Ohm's law). Knowledge of the anatomy and of the highly pressurized system make it possible to predict alternative communicating routes that allow the redistribution of pressure within the system. The most common pathways are gastroesophageal collaterals, paraesophageal varices, gastrorenal shunts, splenorenal shunts, and the recanalized umbilical vein^(12)^.

## CHARACTERISTICS OF SHUNTS

**Figure 3:**
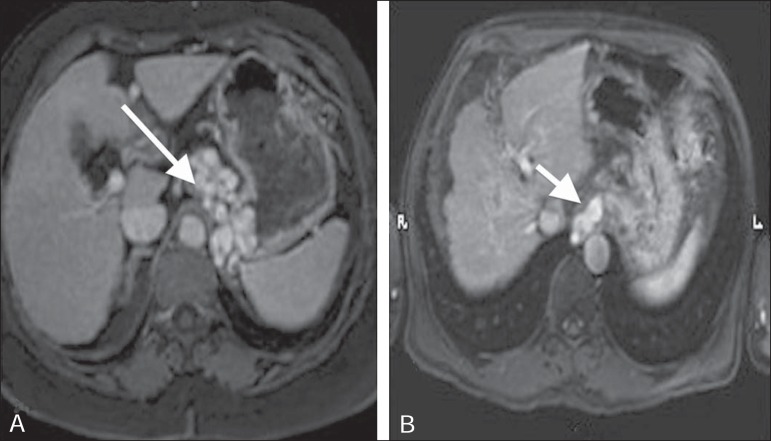
**A,B:** Contrast-enhanced, T1-weighted magnetic resonance imaging of the abdomen, in the axial plane, showing portal hypertension. Note the perigastric varices (arrow in **A**) and paraesophageal varices (arrow in **B**).

**Figure 4:**
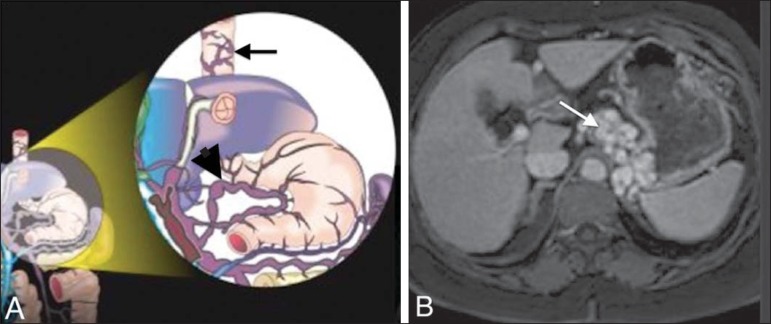
**A:** Schematic drawing showing the anatomy of the left gastric vein (arrowhead). **B:** Contrast-enhanced, T1-weighted magnetic resonance imaging, in the axial plane, of varices along the gastric wall. Note the esophageal vessels that form the esophageal varices (arrow in **A**).

The left gastric vein, formerly known as the gastric coronary vein, is the most commonly detected portosystemic collateral^(12)^ (Figures 3A and 4). It drains the anterior and posterior surfaces of the stomach and ascends via the lesser curvature of the stomach and the esophageal hiatus, where it receives the esophageal veins, continuing its course inferiorly, terminating at the right portal vein. A left gastric vein diameter > 6 mm suggests portal hypertension. The left gastric vein has two branches: the anterior (Figure 4A), which gives rise to esophageal varices, and the posterior (Figure 3B), which gives rise to the paraesophageal veins^(7)^. There is rarely any communication between the anterior and posterior branches.

**Figure 5:**
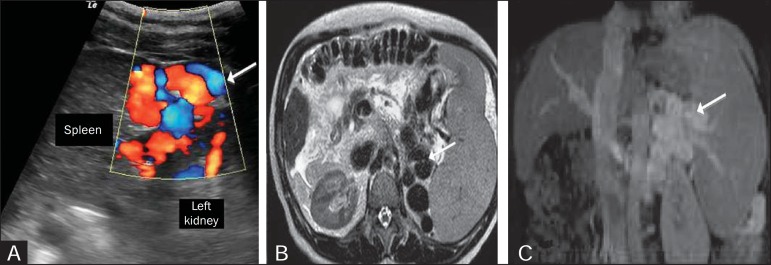
**A:** Longitudinal ultrasound and Doppler flow study showing the presence of large caliber, tortuous vessels (arrows) between the left kidney and the spleen. **B:** T2-weighted magnetic resonance imaging showing large caliber vessels represented by tortuous serpentine structures that exhibit T2 low signal intensity, located between the spleen and the left kidney (arrow). **C:** Contrast-enhanced, T1-weighted magnetic resonance imaging, in the coronal plane, showing the shunt (arrow).

**Figure 6:**
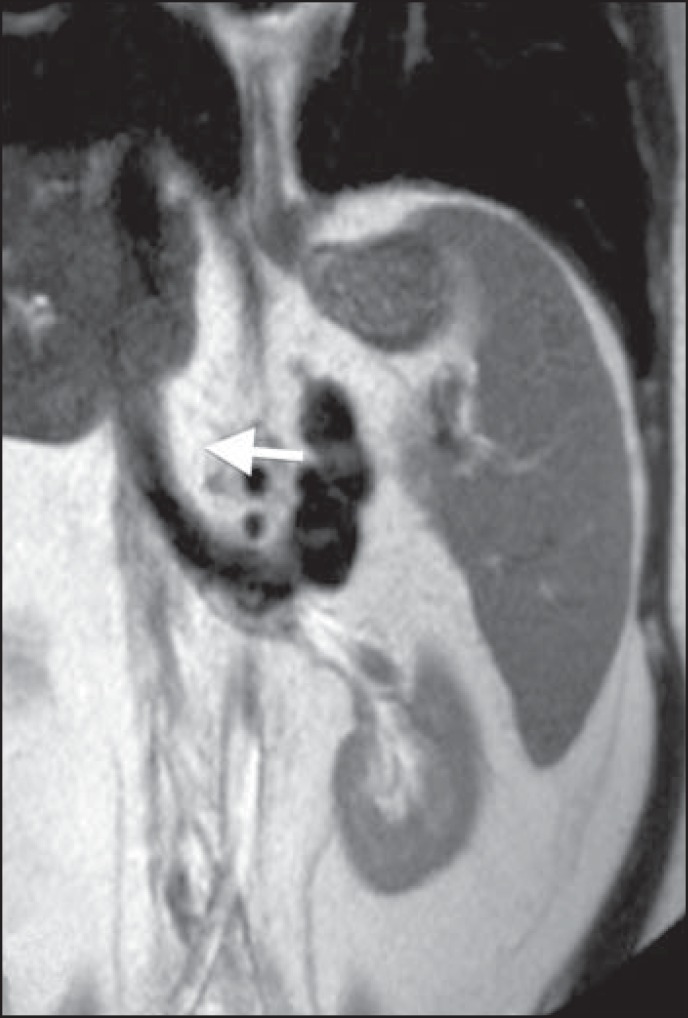
Splenorenal shunt in a patient with hepatitis C (arrow).

In normal physiology, there is a communicating vein between the splenic vein and the left renal vein. That communication can be mediated by the left gastric vein, the posterior gastric vein, the short gastric veins, or other tributaries of the splenic vein (Figures 5 and 6). The collaterals can have an indefinite course-anterior pararenal, posterior pararenal spaces or crossing the adrenal space^(7)^.

**Figure 7:**
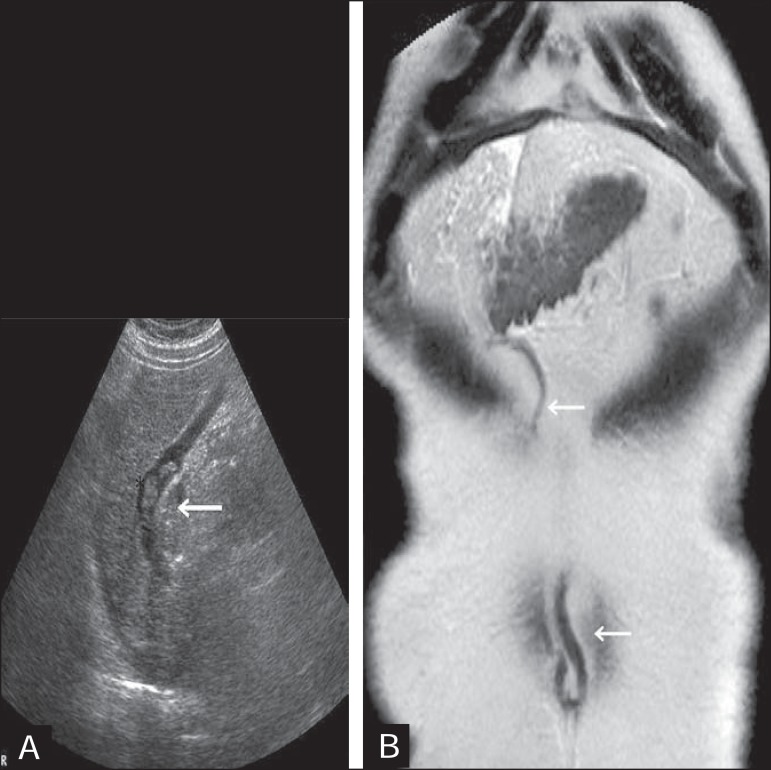
**A:** Longitudinal view from B-mode ultrasound showing the recanalized paraumbilical vein partially filled with hypoechoic material, forming a partial thrombus **B:** T2-weighted magnetic resonance imaging, in the coronal plane, showing the entire trajectory of the paraumbilical vein, from its origin at the round ligament to the umbilicus (arrow).

A recanalized umbilical vein can occur in the context of portal hypertension, traveling along the falciform or round ligament^(13)^, as can be seen in Figure 7. The collateral system can drain into the superior epigastric vein or the internal thoracic vein, draining as far as the superior vena cava, or optionally, into the inferior epigastric vein to the external iliac veins^(14)^.

Other, less common, portosystemic shunts can be seen, such as collaterals of the gallbladder wall (Sharpey's plexus)^(15)^, shunts from the superior mesenteric vein to the right renal vein, mesenteric varices, and diaphragmatic collaterals.

**Figure 8:**
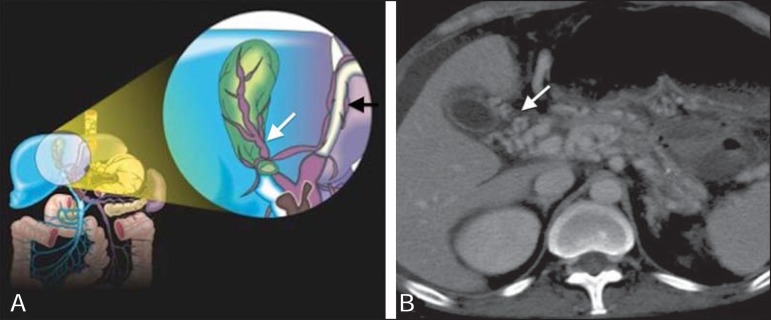
**A:** Schematic drawing showing the cystic and pericholecystic veins in the wall of the gallbladder (white arrow). **B:** Contrast-enhanced axial tomography of the abdomen showing cystic and pericholecystic varices along the wall of the gallbladder (arrow). Note also the vessels along the paraumbilical vein (black arrow in **A**), as demonstrated in Figure 7.

Varicosities of the gallbladder wall correspond to the communication between the cystic vein (portal circulation) and the vessels of the abdominal wall (systemic circulation)^(16)^. Such varicosities are usually identified after thrombosis of the portal vein with cavernous transformation (Figure 8).

**Figure 9:**
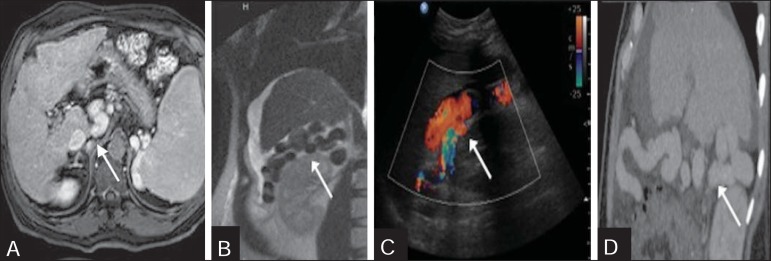
**A:** Contrast-enhanced, T1-weighted magnetic resonance imaging revealing a rare shunt characterized by the presence of dilated, tortuous vessels alongside the renal capsule (arrow). **B:** T2-weighted magnetic resonance imaging, in the coronal plane, showing the same shunt, represented by vessels with low signal intensity (arrow). **C,D:** Longitudinal views from Doppler flow studies and a sagittal reformatted computed tomography scan.

Anastomoses between the superior mesenteric vein and renal capsular veins are rare shunts, of uncertain pathophysiology, which drain into the right renal vein, inferior vena cava, and systemic circulation. In the various imaging modalities, anastomoses can be identified by the presence of dilated, tortuous vessels alongside the renal capsule (Figure 9).

**Figure 10:**
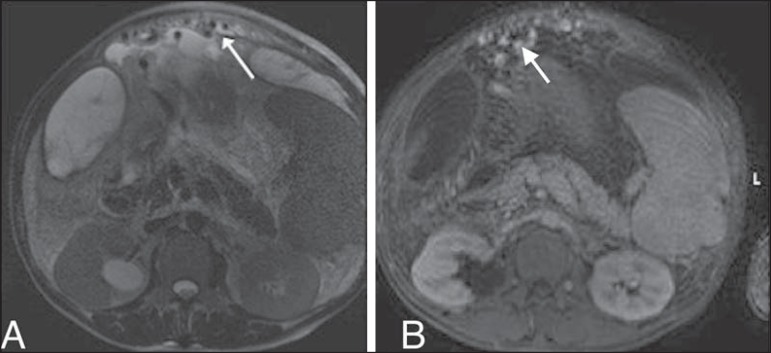
T2-weighted magnetic resonance imaging (**A**) and contrast-enhanced, T1-weighted magnetic resonance imaging (**B**), showing dilated, tortuous mesenteric varices (arrows), with probable drainage to the gonadal vein, renal vein, or inferior vena cava.

The mesenteric varices are also uncommon collaterals^(16)^ that create communication between the intestinal tract or the superior/inferior retroperitoneal tributaries and the systemic circulation (gonadal vein, renal vein, or inferior vena cava), as depicted in Figure 10.

In a left diaphragmatic shunt, the vessel originates from a left peripheral branch of the portal vein and communicates with the inferior phrenic vein at the level of the triangular ligament, subsequently draining into the left renal vein or inferior vena cava^(16)^. In a right diaphragmatic shunt, the vessel originates from a medial peripheral branch of the portal vein and crosses to the diaphragmatic surface of the liver, creating an anastomosis with the internal thoracic vein or intercostal veins.

## CONCLUSION

Portal hypertension has a variety of presentations, as do portosystemic shunts in imaging studies. Radiologists should be prepared for the various presentations of such shunts, reporting their characteristics objectively, which will facilitate the diagnostic investigation and treatment of these anomalies.
